# Cerebral Blood Flow and Brain Functional Connectivity Changes in Older Adults Participating in a Mindfulness-Based Stress Reduction Program

**DOI:** 10.3390/bs12020048

**Published:** 2022-02-14

**Authors:** Aleeze Sattar Moss, Diane K. Reibel, Nancy Wintering, Faezeh Vedaei, Hannah Porter, Mohsen Khosravi, Justin Heholt, Mahdi Alizadeh, Feroze B. Mohamed, Andrew B. Newberg

**Affiliations:** 1 Department of Integrative Medicine and Nutritional Sciences, Thomas Jefferson University, Philadelphia, PA 19107, USA; aleeze.moss@jefferson.edu (A.S.M.); diane.reibel@jefferson.edu (D.K.R.); nancy.wintering@jefferson.edu (N.W.); hannah.porter@jefferson.edu (H.P.); mohsen.khosravi@jefferson.edu (M.K.); 2 Department of Radiology, Thomas Jefferson University, Philadelphia, PA 19107, USA; faezeh.vedaei@jefferson.edu (F.V.); mahdi.alizadeh2@jefferson.edu (M.A.); feroze.mohamed@jefferson.edu (F.B.M.); 3 Department of Surgery, Allegheny General Hospital, Pittsburgh, PA 15212, USA; justin.heholt@ahn.org

**Keywords:** mindfulness, functional connectivity, cerebral blood flow, elderly, depression, brain

## Abstract

There is a growing interest in the potential beneficial effects of mindfulness meditation training in protecting against age-related physical, emotional, and cognitive decline. The current prospective, single-center, single-arm study investigated if functional magnetic resonance imaging-based changes in cerebral blood flow and brain functional connectivity could be observed in 11 elderly adults (mean age 79) after participation in a Mindfulness-Based Stress Reduction (MBSR) program. The results showed significantly (*p* < 0.05) altered cerebral blood flow and functional connectivity in the cingulate gyrus, limbic structures, and subregions of the temporal and frontal lobes, similar to findings of other meditation-related studies in younger populations. Furthermore, these changes were also associated with significant improvements in depression symptoms. This study suggests that the MBSR program can potentially modify cerebral blood flow and connectivity in this population.

## 1. Introduction

The growing population of older adults faces increasing stressors as they age [1]. Many live with multiple chronic medical conditions and often chronic pain, which is associated with considerable psychological distress and physical disability [2]. Challenged by chronic illness, loss of loved ones, social isolation, and declining physical function, varying degrees of anxiety and depression are common among the elderly and are associated with increased disability and decreased well-being [3]. Research on MBSR specifically with older adults has demonstrated a number of beneficial effects, including reductions in anxiety, depression, and loneliness [4,5]. Improvements in cognitive function have also been shown [6,7,8,9]. In most of these studies with MBSR in the elderly, the mean age has been 65 to 75 years.

There are few studies exploring the neurophysiological changes that mindfulness training may produce in elderly individuals. In the past, it was thought that the elderly are less able to alter brain functions in beneficial ways. For example, as people age, they are less likely to form new neuronal connections and have less neuroplasticity [10]. Cerebral blood flow as well as functional connectivity (measuring how different parts of the brain work together) have also been shown to decrease with aging, particularly in areas associated with cognition [11,12].

However, a number of recent studies have suggested that neuroplasticity is still possible in the elderly and that environmental influences may augment neuronal connections and function [13,14,15]. The question is whether elderly individuals might experience changes in these neurophysiological parameters as the result of participating in an MBSR program and whether such changes might be associated with reduced anxiety and/or depression. 

The MBSR program centers on the practice of mindfulness meditation, which involves the cultivation of an open, curious, and nonjudgmental awareness of present-moment experience [16]. The MBSR curriculum includes training in mindfulness meditation through different formal practices, including body scan meditation, sitting meditation, mindful stretching, mindful walking and eating, as well as mindful communication practices. The program also includes didactic information on stress physiology and healthy responding versus reacting to stress. MBSR was originally designed for a heterogeneous patient population, but most studies have been on younger populations including generally healthy individuals younger than 65, patients with selected medical conditions, and even students such as college, medical, or nursing students [17,18].

The MBSR program for this current study was modified for elderly individuals, and this has been reported previously [19]. While several studies have explored the physiological and clinical effect of MBSR in older adults, only a few have specifically explored the neurophysiological correlates of such improvements. Our goal was to determine if we can observe changes in cerebral blood flow or functional connectivity in these elderly individuals as the result of the MBSR program and if such changes are similar to or distinct from those observed in studies of younger populations.

Importantly, in this current study, we used fMRI to assess changes in cerebral blood flow (CBF) using arterial spin labeling (ASL) and in functional connectivity using resting-state Blood Oxygen Level-Dependent (BOLD) scans in elderly subjects before and after participating in an 8-week MBSR program. ASL fMRI imaging allows for measurement of CBF in various states. ASL fMRI has not been used extensively to study mindfulness or other forms of meditation. One small study of 15 meditation-naïve subjects using ASL fMRI found that anxiety reduction resulting from mindfulness meditation was associated with activation of the anterior cingulate cortex, ventromedial prefrontal cortex, and anterior insula, areas involved in self-referential thought [20]. A more recent small study using these techniques with 10 meditation-naïve subjects found that focused attention meditation was positively associated with changes of resting-state functional connectivity between the default mode network (DMN) and the dorsal attention network (DAN), between the DMN and the insula, and between the DAN and the frontoparietal control network (FPN) [21]. The same study showed that meditation training was negatively associated with changes of resting-state functional connectivity between the DMN and the FPN and between the DAN and the brain’s visual regions.

For the current study, we performed resting BOLD fMRI scans before and after an MBSR program to assess functional connectivity changes in elderly subjects. In addition, ASL scans were performed pre and post the MBSR program to more directly measure changes in CBF. During each imaging session, we performed two ASL scans—one in the resting condition and one during an actual meditation practice called “body scan” in which subjects follow an audio file which guides them through the practice in which they focus their attention on any sensations in their body. This is a common meditation practice that is part of the MBSR program, and thus, we hoped this study would help demonstrate whether we can show changes between meditation and non-meditation states and whether these states are altered by participation in the 8-week MBSR program.

## 2. Materials and Methods

### 2.1. Participants

Subjects were recruited from an Independent Living arm of a Life Plan Community (formerly known as a Continuing Care Retirement Community or CCRC) to participate in an 8-week MBSR program. Recruitment was announced by the research team in several separate group presentations at the Life Plan Community. An overview of the study elements was provided, and participants were asked if they had any question and if they were interested in participating. After this initial orientation session in which details about the MBSR program and fMRI research study were described, the subjects were invited to volunteer to participate in the fMRI study. A total of 20 subjects who volunteered to participate in the fMRI study met individually with a researcher to be screened in order to determine if they met the inclusion criteria: age > 65 years and Mini-Mental Status Exam score > 25; no prior experience with the MBSR program; no metal in their body (pacemaker, types of stents or ports); no medical condition that could interfere with cerebral blood flow; not on any medication that could affect brain physiology or cerebral blood flow.

Eleven subjects who met all inclusion criteria participated in the study (see Table 1). All participants were Caucasian and were highly educated, with a minimum of a college degree. All participants provided a written informed consent that was approved by the Thomas Jefferson University Institutional Review Board. All subjects participated in an 8-week MBSR program and completed fMRI scans within 2 weeks prior to the start date of the MBSR program (pre-MBSR scan) and again within 2 weeks after completing the MBSR program (post-MBSR scan). Data were acquired from September 2016 to September 2017.

### 2.2. The MBSR Program

The 8-week MBSR was taught by a certified MBSR teacher (certified through the Center for Mindfulness at University of Massachusetts Medical School) with over 25 years of experience specifically using the MBSR curriculum as developed by Jon Kabat-Zinn [16] with some adaptations (see below). MBSR was run on site at the CCRC. Participants met in a community room dedicated each week for the program. The environment had natural lighting and comfortable chairs and was easily accessible. Based on input from the administrators at the CCRC who were aware of residents’ schedules and best time of day to run the program, classes were run in the afternoon (1:30–3:30 pm). During each group session, the instructor led participants in guided mindfulness meditation exercises, mindful yoga, and group discussions with the intent to foster mindful awareness of one’s moment-to-moment experience. Between sessions, participants were asked to practice daily using a CD of guided meditations. Attendance was taken at each session.

The standard MBSR program is an 8-week program that meets once a week for two and a half hours and includes a full day of practice. Participants are asked to practice at home for 45 min a day, listening to guided meditations. The following modifications were made to the standard MBSR program in order to make it more accessible to this population of older adults. The 8-week program for older adults met once a week for 2 h, without a full day retreat. Participants were asked to practice at home for 25 to 30 min daily. The sequence of practices was the same as that of the standard MBSR program including body scan awareness practice, sitting meditation with awareness of breath, mindful yoga, mindful walking, and loving-kindness meditation [16]. Specifically, the MBSR program used in this study comprised the following classes and topics: Class 1: Introduction to mindfulness, attitudinal foundations, breath as a mirror of mind and emotion, identifying present-level stress, and introducing the Body Scan; Class 2: Physiology of stress, perception and creative responding, cultivating mindfulness in daily activities, and introducing Awareness of Breath Meditation; Class 3: The power of being present, meditation in stillness and movement, introducing Mindful Chair Yoga; Class 4: Awareness of stress reactivity, cultivating strength, balance, and flexibility, and introducing Mindful Walking; Class 5: Responding vs. reacting to stress, moving from habitual behaviors to more effective responses, and introducing Expanding Awareness Meditation; Class 6: Interpersonal communication skills and introducing Mindful Listening Practice; Class 7: Kindness and compassion for self and others and introducing Loving-Kindness Meditation; Class 8: Continuing to cultivate mindfulness in day-to-day life and setting intentions for moving forward.

To account for potentially decreased mobility in this elderly population, the yoga practice was adapted to chair yoga instead of floor yoga. Similarly, with mindful walking, participants had the option of walking close to a wall or to hold on to the backs of chairs in case they needed additional support. Each home practice was put on a separate CD, so participants could more easily manage the technology required with playing the CD. Detailed instructions were also given to participants who wanted to listen to recordings as mp3s on their computers or phones. The binder contents were also written in large print for better visibility.

### 2.3. Clinical Measures

Subjects were evaluated for anxiety and depression pre- and post-MBSR program using the Spielberger State/Trait Anxiety score and the Beck Depression Index (BDI) score. Since these data were nonparametric, a Wilcoxon signed-rank test was performed to determine if there were any measurable effects. 

### 2.4. Imaging Acquisition 

The MR imaging was performed on a 3T Siemens mMR PET–MRI scanner using a standard 12-channel head coil. The total scan time was approximately 40 min. Initially, structural MRI brain images were collected using a T1-weighted Magnetization-Prepared Rapid Gradient Echo (MPRAGE) sequence. The imaging parameters used were: FOV = 25.2 cm^2^, voxel size = 0.5 × 0.5 × 1.0 mm^3^, matrix size = 256 × 240, TR = 1600 ms, TE = 2.46 ms, slice thickness = 1 mm, number of slices = 176, flip angle = 9°, and acquisition time = 280 s. Next, resting-state BOLD fMRI data were collected using an Echo Planar Imaging (EPI) sequence. The following imaging parameters were used: FOV = 23.6 cm^2^, voxel size = 3 × 3 × 4 mm^3^, matrix size = 128 × 128, TR = 2000 ms, TE = 30 ms, slice thickness = 4 mm, number of slices = 34, number of volumes = 180, and acquisition time = 360 s. During resting-state fMRI, the subjects were instructed to close their eyes, keep their heads still, and relax for 5 min.

Finally, the subjects underwent two ASL scans. The first scan was performed while the subjects were at rest with their eyes closed as in the resting-state BOLD scan for approximately 6 min. The second scan was performed while participants were guided through “body scan meditation” in which they were instructed to attend to sensations in the body, progressively focusing on different body parts. This is a core meditation practice in the MBSR program. This procedure was followed again post-MBSR.

ASL scans pre- and post-MBSR program were performed using Echo Planar Imaging (EPI) sequence parameters as follows: TR = 2500 ms, TE = 11 ms, FOV = 19.2 cm^2^, voxel size = 3 × 3 × 6 mm^3^. An asymmetrical pulsed ASL sequence (PICORE Q2T) [11] was utilized with the following parameters: 16 slices acquired in ascending order, slice thickness = 6 mm, Bolus Duration = 1675 ms, Inversion/Delay Time = 1800 ms, Labeling time = 700 ms, and labeling pulse flip angle = 90°. In total, 119 volumes were acquired, with the 1st volume containing the M0 image, and the remaining volumes containing 59 pairs of control/labelled images. 

### 2.5. Cerebral Blood Flow Analysis

Preprocessing and statistical analysis were performed on all subjects using Matlab (ver. R2018a), using the standard steps that are incorporated as part of a validated SPM12-based ASL perfusion MRI data processing toolbox, ASLtbx [22]. The processing steps are as follows: (1) Realignment: rigid body spatial transformation was applied to the PASL control and label images in order to remove motion artifacts in the PASL time-series; (2) Co-Registration: the motion-corrected PASL images and the M0 images were co-registered to the structural T1 space; (3) Smoothing: the PASL images were smoothed before CBF calculation to prevent noise propagation and to improve the signal-to-noise ratio (SNR). A 6 mm full-width at half maximum (FWHM) Gaussian kernel was used; (4) Cerebral Blood Flow Calculation: perfusion difference images were generated, and the mean whole brain cerebral blood flow (mL/100 g/min) was calculated for each subject by pairwise control–label subtraction using the established one compartment model [23,24]. CBF was estimated using the following formula and parameters:

$$f=\frac{\Delta M\lambda R_{1a}\cdot \exp(\omega R_{1a})}{2M_{0}\alpha }\;\cdot {\left[1-\exp(-\tau \cdot R_{1a})\right]}^{-1}$$

where *f* is CBF, Δ*M* is the difference signal between the control and the label acquisitions, *R*_1*a*_ is the longitudinal relaxation rate of blood, *τ* is the labeling time, *ω* is the post-labeling delay time, *α* is the labeling efficiency, *λ* is blood/tissue water partition coefficient, and *M*_0_ is approximated by the control image intensity. The parameters used in this study were *R*_1*a*_ = 0.67 s^−1^, *α* = 0.95, *λ* = 0.9 g/mL, *τ* = 0.7 s, *ω* = 1.8 s; (5) Segmentation: the anatomical images were divided into individual tissue types (grey matter, white matter, and CSF) for use in the outlier cleaning step. The high-resolution structural image (T1-MPRAGE) were used and compared to prior established tissue probability maps generated in SPM; (6) Outlier cleaning: slice-wise outlier cleaning was applied to the perfusion difference images produced in step 4 and utilized the segmentations produced in step 5 [25]; (7) Normalization: deformations produced from the SPM12 segmentation implemented in step 5 were applied to the outlier-cleaned CBF maps to transform the functional data into the standard Montreal Neurological Image (MNI) space.

After pre-processing was completed, a quantitative group analysis was performed using SPM12 software. This method involved statistical analyses of the normalized, smoothed, outlier-corrected perfusion images in order to assess the group differences in resting-state cerebral blood flow before MBSR and after it. In order to find this effect, the resting condition and the ‘body scan’ condition were compared using a 2 × 2 full factorial ANOVA design, factor 1 was the condition (resting or body scan), and factor 2 was time (pre MBSR and post MBSR). This was a voxel-based analysis performed on the whole brain, using a T-contrast reflecting the mean difference between the two time periods and the variation within them. A post hoc FDR correction was applied based on the regions we hypothesized would be involved in the meditation process. The regions that survived FDR correction for multiple comparisons with a *p* value < 0.05 were identified. These regions represent the change in CBF after two months of participating in the MBSR program seen during the resting condition and during body scan meditation.

### 2.6. Functional Connectivity Analysis

Preprocessing was completed in SPM12 (UCL, London, UK) within the MATLAB^®^ environment (MathWorks, Natick, MA, USA). Functional data were realigned, with the mean image co-registered to structural volumes. Segmentation of structural volumes in grey matter, white matter, and CSF spaces was completed, and volumes were spatially normalized to the Montreal Neurological Institute (MNI) space. Finally, normalized images were smoothed temporally. Functional data were denoised using a component-based noise correction method (CompCor) within the CONN toolbox (Neuroimaging Informatics Tools and Resources Clearinghouse, National Institutes of Health, Bethesda, MD, USA). The structural volumes were separated into white matter, gray matter, and CSF confounds. A band-pass filter with a range of 0.008 to 0.09 Hz was applied to the data to restrict the analysis to a limited frequency window, while white matter and CSF confounds were placed in a 3D space. This analysis pipeline is well established and widely used in the fMRI community for evaluating resting-state data.

Next, a seed-based analysis was carried out on this analyzed dataset. Regions of interest (ROIs) were defined using the Harvard-Oxford (Center for Morphometric Analysis, Massachusetts General Hospital-East, Charlestown, MA, USA) atlas. Changes in functional connectivity between several areas of interest, including anterior cingulate cortex, amygdala, cerebellum, limbic system, parahippocampus, and prefrontal cortex, were examined with both ROI-to-voxel and ROI-to-ROI analyses. General linear models (GLMs) were used to compare resting-state connectivity between pre- and post-MBSR. A *p* < 0.01 uncorrected height threshold and *p* < 0.05 false discovery rate (FDR)-corrected cluster threshold were used along with both single and two-tailed correlations.

## 3. Results

### 3.1. Study Completion Rate, MBSR Attendance, and Clinical Response 

All of the 11 subjects who participated in this fMRI study completed the 8-week MBSR program and both the pre- and post-program brain scans. Nine of the subjects attended all eight MBSR classes, and two attended seven classes.

When evaluating clinical response in this small cohort, there was a significant decrease in BDI scores using the Wilcoxin signed-rank test with a median pre-MBSR score of 2.5 (interquartile range of 6.0) and a median post-MBSR score of 1.0 (interquartile range of 3.0) (*p* < 0.05), with a Cohen’s d of 0.51 and a non-significant decrease in state anxiety (see Table 1). No subjects reported any adverse events or experiences during the MBSR program or during procedures such as the MRI. This included no reports of increased anxiety, stress, or depression.

### 3.2. Changes in Cerebral Blood Flow

Changes in CBF between the pre- and post-MBSR states are shown in Table 2 and Table 3. Overall, during the resting state, there were significant increases in the post-MBSR program scan compared to the pre-MBSR program scan in the right PFC, anterior and posterior cingulate gyrus, left superior temporal gyrus, and left superior frontal gyrus. During the meditation state, there were significant increases in the post-MBSR program compared to the pre-MBSR program scan in the left PFC, left putamen, posterior cingulate gyrus, and left and right inferior parietal lobes.

### 3.3. Changes in Functional Connectivity

The functional connectivity results are provided in Table 4 and described in more detail below. ROI-to-voxel analysis showed increased connectivity between the anterior cingulate cortex (ACC) and the left insular cortex, posterior cingulate cortex, putamen, and left orbitofrontal cortex (FDR-corrected *p* = 0.002). ROI-to-voxel analysis showed decreased connectivity between the ACC and the cerebellum, left inferior temporal gyrus, and left superior temporal gyrus (FDR-corrected *p* = 0.008).

ROI-to-voxel analysis showed increased connectivity between the amygdala and the right parahippocampal cortex, right inferior temporal gyrus, right orbitofrontal cortex, and right hippocampus (FDR-corrected *p* = 0.02). There were no significant areas of decreased connectivity using the amygdala as the ROI.

ROI-to-voxel analysis showed increased connectivity between the parahippocampus and the right hippocampus, right insular cortex, right inferior temporal gyrus, and cerebellum (FDR-corrected *p* = 0.00006). There were no significant areas of decreased connectivity using the parahippocampus as the ROI. 

ROI-to-voxel analysis showed increased connectivity between the prefrontal cortex (PFC) and the right superior temporal gyrus and right inferior temporal gyrus (FDR-corrected *p* = 0.0002). There were no significant areas of decreased connectivity using the PFC as the ROI.

ROI-to-ROI analysis showed increased connectivity between the right amygdala and the left hippocampus (FDR-corrected *p* = 0.033), between the right hippocampus and the right parahippocampus (FDR-corrected *p* = 0.039), and between the left and the right caudate nuclei (FDR-corrected *p* = 0.0238).

## 4. Discussion

Neuroimaging studies of mindfulness have grown exponentially in the past decade and demonstrate that mindfulness training can change both brain structure and brain function. A recent systematic review of 21 studies indicates that the emotional and behavioral changes observed in people after participating in Mindfulness-Based Interventions (MBIs) are related to both functional and structural changes in the brain [26]. This review article reported that participation in MBIs is associated with increased activity, connectivity, and volume in the prefrontal cortex, the cingulate cortex, the insula, and the hippocampus. In addition, participation in MBIs is associated with increased amygdala functional connectivity with the prefrontal cortex and earlier deactivation after exposure to emotional stimuli.

A separate study explored the MBSR course in 140 healthy adults with an average age of 44.3 years randomized to the MBSR program, an active control, or a waitlist control group [27]. The study found increased resting connectivity between the executive control network (dorsolateral PFC) and the default mode network (posterior cingulate cortex) for those participating in the MBSR relative to the control groups. Further, the MBSR participants showed a significantly stronger relationship between days of practice and increased connectivity between these structures.

Smart et al. [28] found that mindfulness training with older adults with subjective cognitive decline led to decreases in memory complaints, increases in memory efficacy, and increased total brain volume. Another earlier study by Moynihan et al. [29] found that MBSR produced small but significant changes in executive function and sustained left frontal asymmetry of the EEG alpha band in older adults. A study by Malinowski and colleagues [30] demonstrated improvements in goal-directed visuospatial attention and associated electrophysiological changes in older adults with mindfulness training. In the aforementioned studies of MBSR with older adults, the mean age ranged between 65 and 75 years.

Additionally, in a recent randomized controlled trial of 146 older individuals, mindfulness training was compared to a cognitive fitness program to determine brain effects and also effects on cognition [31]. Mindfulness training was associated with greater improvements in cognition that were associated with increased connectivity within the default mode network, particularly between the right hippocampus and the posteromedial cortex and between the left hippocampus and the lateral parietal cortex. These findings are important since the default mode network is associated with cognitive function and is particularly vulnerable to aging affects.

This current single-arm study enrolled one of the oldest cohorts of subjects studied with fMRI to evaluate cerebral blood flow and functional connectivity before and after an MBSR program (the mean age of the subset of the study participants was 79 years). The study showed that conducting neuroimaging with this population is feasible and can demonstrate significant changes even in a small sample. Several significant changes in CBF and functional connectivity associated with participation in the MBSR program were observed, consistent with a number of the above-mentioned findings in other studies of MBIs [20,21]. Cerebral blood flow was found to be increased both at rest and during meditation after participating in the MBSR program. During the resting scans, there was increased CBF in the right PFC, anterior cingulate gyrus, left superior temporal gyrus, left superior frontal gyrus, posterior cingulate, and right anterior cingulate. These are important findings, since they suggest changes in the basic function of the brain as the result of participating in the MBSR program. Such changes may contribute to the reductions in depressive symptoms that we observed in our subjects. In fact, a recent study of transcranial magnetic stimulation with intermittent theta bursts to the prefrontal cortex significantly reduced depression symptoms [32]. These changes have also been linked to reduced anxiety, which we did not observe to a significant degree in this study. During the body scan meditation itself, there were significant increases in CBF in the left PFC, the left putamen, the posterior cingulate gyrus, and left and right inferior parietal lobes. The finding of changes in the brain during the body scan meditation before and after undergoing the MBSR program suggest a training effect, such that practicing meditation over a period of time changes the way in which the brain actually becomes active during the practice. Future studies with a larger number of subjects will be needed to better understand the relationship between longitudinal changes in CBF during meditation itself and changes in brain function at rest that might relate to clinical effects.

In the current study, there was generally increased connectivity between brain structures involved in emotional self-regulation such as the anterior cingulate cortex, prefrontal cortex, cerebellum, caudate nuclei, and limbic regions. Also observed was increased connectivity between specific regions, such as the amygdala and the hippocampus, and the amygdala and parahippocampus. These results suggest that there are functional changes, particularly, increased connectivity in areas associated with emotion regulation, attention, and memory in older adults participating in the MBSR program.

The findings are consistent with prior studies in younger populations in which the prefrontal cortex, limbic areas such as the hippocampus and the amygdala, and the posterior cingulate are affected by mindfulness programs. For example, a small study of 14 patients with mild cognitive impairment (MCI) participating in MBSR revealed increased functional connectivity between the posterior cingulate cortex and the bilateral medial prefrontal cortex and left hippocampus compared to controls [33]. These results indicated that in adults with MCI, MBSR may have a positive impact on the regions of the brain most related to MCI. In another study, 35 adults presenting with psychological distress underwent a 3-day mindfulness program and were found to have increased resting-state functional connectivity between brain regions associated with executive function [34]. Decreased resting-state functional connectivity in the amygdala–anterior cingulate cortex were also found after the 3-day mindfulness program, suggesting a pathway for the stress reduction effects of mindfulness [35]. Finally, an fMRI study by Yang et al. [36] of 13 novices with a mean age of 24 who underwent 40 days of mindfulness training showed increased connectivity between the precuneus and the temporoparietal junction, but reduced functional connectivity in the frontal brain regions. This latter finding is in the opposite direction of our findings, and future studies will be needed to determine whether these changes are distinct among differently aged patient populations.

The current study supports these previous reports of neuroplastic changes with mindfulness training and adds to the growing field by observing changes in elderly adults. Our study results suggest that the MBSR program alters functional connectivity in emotional networks in older individuals. There is also overlap, with some of these regions having both altered functional connectivity as well as altered CBF. These areas include the PFC, anterior cingulate cortex, and posterior cingulate cortex, all areas involved in attentional control and emotional regulation.

The study limitations include a small sample size and lack of diversity in the population of older adults, which can limit the generalizability of our findings. In addition, this single-arm fMRI study did not include a control group and thus did not control for factors such as time and attention. More research including randomized trials with larger sample sizes, active control groups, and long-term follow-up is needed to further investigate the promising results of this study.

## Figures and Tables

**Table 1:** Patient demographics and pre/post clinical measures.

Characteristic	Measure/Score
Age	79.0 ± 5.2 years old (range 71–87 years)
Gender	9 females and 2 males
Education	18 ± 3 years
Beck Depression Index Pre	2.5 (interquartile range of 6.0)
Beck Depression Index Post	1.0 (interquartile range of 3.0)
State Anxiety Pre	29.0 (interquartile range of 9.5)
State Anxiety Post	31.5 (interquartile range of 7.0)

**Table 2:** Cerebral blood flow differences between the pre- and the post- MBSR program scans during the resting state (PFC = prefrontal cortex, ACC = anterior cingulate cortex). The peak intensity represents the T statistic at the peak statistical significance for the cluster.

Region	PEAK MNI Coordinate	Peak Intensity	Peak Level *p* Value Uncorrected	Peak Level *p* Value FDR Corrected
Right PFC	32 38 –4	4.43	0.001	0.03
ACC	–6 28 –12	3.30	0.001	0.02
Left Superior Frontal Gyrus	–22 48 48	3.60	0.001	0.01
Posterior Cingulate	–14 –66 8	3.34	0.001	0.01
Left Superior Temporal Gyrus	–62 –28 4	3.12	0.002	0.01
Right Anterior Cingulate	18 26 28	2.77	0.004	0.02

**Table 3:** Cerebral blood flow differences between the pre- and the post- MBSR program scans during the meditation state (PFC = prefrontal cortex). The peak intensity represents the T statistic at the peak statistical significance for the cluster.

Region.	PEAK MNI Coordinate	Peak Intensity	Peak Level *p* Value Uncorrected	Peak Level *p* Value FDR Corrected
Left PFC	–4 54 –8	3.24	0.001	0.03
Posterior Cingulate	–12 –64 12	4.32	0.001	0.02
Right Superior Temporal Lobule	36 6 –24	–3.52	0.001	0.01
Left Putamen	–22 4 –4	3.18	0.002	0.01
Left Inferior Parietal Lobule	–50 –30 22	3.03	0.002	0.01

**Table 4:** Coordinates of the largest cluster size for each respective ROI for functional connectivity analysis (ACC = anterior cingulate cortex, PFC = prefrontal cortex).

Region	PEAK MNI Coordinate	Cluster Size (Voxels)	FDR Corrected *p* Value
ACC	–12 –38 46	842	0.002
Amygdala	26 12 –44	603	0.019
Cerebellum	–12 –74 –18	654	0.017
Parahippocampus	46 –62 2	1301	0.001
PFC	64 –10 –24	684	0.011

## Data Availability

Data from this study are available upon request.
